# Designing Multi-arm Multistage Adaptive Trials for Neuroprotection in Progressive Multiple Sclerosis

**DOI:** 10.1212/WNL.0000000000200604

**Published:** 2022-05-03

**Authors:** Vivien Li, Baptiste Leurent, Frederik Barkhof, Marie Braisher, Fay Cafferty, Olga Ciccarelli, Arman Eshaghi, Emma Gray, Jennifer M. Nicholas, Mahesh Parmar, Guy Peryer, Jenny Robertson, Nigel Stallard, James Wason, Jeremy Chataway

**Affiliations:** From the Florey Institute of Neuroscience and Mental Health (V.L.), University of Melbourne; Department of Neurology (V.L.), Royal Melbourne Hospital, Australia; Department of Medical Statistics (B.L., J.M.N.) and International Statistics and Epidemiology Group (B.L.), London School of Hygiene and Tropical Medicine, UK; Department of Radiology and Nuclear Medicine, Neuroscience Campus Amsterdam (F.B.), VU University Medical Center, Amsterdam, the Netherlands; Queen Square Institute of Neurology and Centre for Medical Image Computing (F.B.), Department of Neuroinflammation, Queen Square Multiple Sclerosis Centre (M.B., O.C.), and NMR Unit, Department of Neuroinflammation (A.E.), Faculty of Brain Sciences, UCL Queen Square Institute of Neurology; MRC Clinical Trials Unit at UCL, Institute of Clinical Trials and Methodology (F.C., M.P., J.C.), and Department of Computer Science, Centre for Medical Image Computing (A.E.), University College London; National Institute for Health Research (F.B., O.C., J.C.), University College London Hospitals Biomedical Research Centre; UK Multiple Sclerosis Society (E.G., G.P., J.R.), London; Faculty of Medicine and Health Sciences (G.P.), University of East Anglia, Norwich; Statistics and Epidemiology, Division of Health Sciences (N.S.), Warwick Medical School, University of Warwick, Coventry; and Population Health Sciences Institute (J.W.), Newcastle University, UK.

## Abstract

There are few treatments shown to slow disability progression in progressive multiple sclerosis (PMS). One challenge has been efficiently testing the pipeline of candidate therapies from preclinical studies in clinical trials. Multi-arm multistage (MAMS) platform trials may accelerate evaluation of new therapies compared to traditional sequential clinical trials. We describe a MAMS design in PMS focusing on selection of interim and final outcome measures, sample size, and statistical considerations. The UK MS Society Expert Consortium for Progression in MS Clinical Trials reviewed recent phase II and III PMS trials to inform interim and final outcome selection and design measures. Simulations were performed to evaluate trial operating characteristics under different treatment effect, recruitment rate, and sample size assumptions. People with MS formed a patient and public involvement group and contributed to the trial design, ensuring it would meet the needs of the MS community. The proposed design evaluates 3 experimental arms compared to a common standard of care arm in 2 stages. Stage 1 (interim) outcome will be whole brain atrophy on MRI at 18 months, assessed for 123 participants per arm. Treatments with sufficient evidence for slowing brain atrophy will continue to the second stage. The stage 2 (final) outcome will be time to 6-month confirmed disability progression, based on a composite clinical score comprising the Expanded Disability Status Scale, Timed 25-Foot Walk test, and 9-Hole Peg Test. To detect a hazard ratio of 0.75 for this primary final outcome with 90% power, 600 participants per arm are required. Assuming one treatment progresses to stage 2, the trial will recruit ≈1,900 participants and last ≈6 years. This is approximately two-thirds the size and half the time of separate 2-arm phase II and III trials. The proposed MAMS trial design will substantially reduce duration and sample size compared to traditional clinical trials, accelerating discovery of effective treatments for PMS. The design was well-received by people with multiple sclerosis. The practical and statistical principles of MAMS trial design may be applicable to other neurodegenerative conditions to facilitate efficient testing of new therapies.

Progressive multiple sclerosis (PMS) is a significant health problem worldwide^1^ and has considerable financial costs for health care systems, patients, and their caregivers, with costs increasing at higher levels of disability.^2-4^ Despite extensive efforts, there are few proven therapies for PMS. Compared to the predominantly inflammatory pathology in relapsing multiple sclerosis (MS) targeted by current treatments, the neurodegenerative processes driving progression in PMS are complex and less well-defined.^5,6^ There is a pipeline of candidate therapies from preclinical studies, but the challenge is testing them efficiently in clinical trials with appropriate outcome measures to determine whether they can successfully slow disability progression.

One potential avenue is improving efficiency of trials by incorporating adaptive elements in a multi-arm multistage (MAMS) platform design. MAMS trials aim to evaluate multiple experimental arms and seamlessly integrate traditional phase II and III evaluations into a single trial. They have been successful in accelerating evaluation of therapies and changing practice in other disease settings, such as cancer^7^ and infectious diseases.^8^ They are also increasingly being considered for neurologic conditions such as Alzheimer disease,^9^ Parkinson disease^10^ and motor neuron disease.^11,12^ These neurodegenerative conditions share commonalities with PMS, where there is a marked translational gap between the relative abundance of early phase trials stemming from increased understanding of disease pathobiology and lack of positive phase III trials leading to disease-modifying treatments.

Adaptive MAMS platform designs offer flexible features that can provide efficiencies at various levels^13^ (Table 1). These include simultaneous evaluation of multiple treatments against a common standard of care, reducing both time and numbers of patients required; the ability to add new treatments as they become relevant, avoiding lengthy setup times for multiple trials; dropping treatments that are not showing sufficient promise, allowing redirection of resources; and incorporation of the traditional separate phase II and III evaluations within a single protocol with seamless transitions.

**Table 1:**
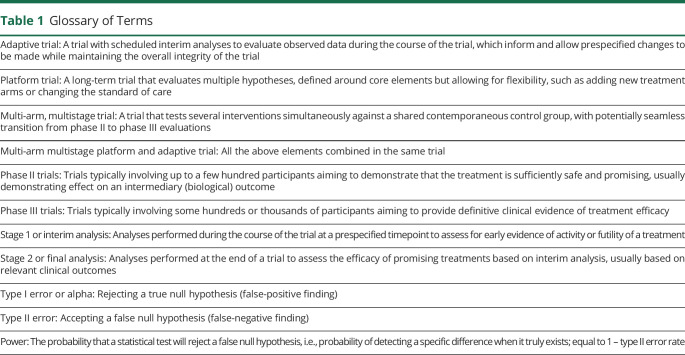
Glossary of Terms

With the aim of designing a MAMS trial in PMS, the UK MS Society Expert Consortium for Progression in MS Clinical Trials set up 4 working groups on outcome measures, trial design, treatment selection, and trial infrastructure. Each group included members with relevant expertise and worked closely with the patient and public involvement (PPI) group throughout the development process. The work of the treatment selection working group on identifying and shortlisting candidate treatments, focusing on licensed drugs that can be repurposed, has been reported elsewhere.^14^

This article describes the work of the trial design and outcome measures working groups. We discuss key elements of the MAMS trial design based on evaluation of 3 candidate treatments against standard of care in 2 analysis stages, including selection of the primary interim and final outcomes, sample size, and other statistical considerations.

## Methods

### Outcome Measures

The outcome measures working group comprised individuals with expertise in MS trials, imaging, and biomarkers, as well as people with MS with lived experience of the condition. The group reviewed the literature to determine outcome measures relevant to a MAMS trial evaluating predominantly neuroprotective treatments. Individual members submitted proposed outcome measures based on their expertise with final prioritization of outcomes determined in consensus meetings.

The MAMS design allows distinct interim (stage 1) and final (stage 2) outcomes. The final outcome should be clinically derived and relevant to patients and regulators. The interim outcome serves as an early indicator of whether a treatment is likely to be effective and hence should be continued into the second stage of the trial while minimizing the likelihood of ceasing truly effective treatments. It should reflect the underlying association between the treatment and the clinical outcome. The absence of effect on the interim outcome should be indicative of the absence of effect on the final outcome, although the converse may not necessarily hold.^15^

### Trial Design

The trial design working group, comprising experts in design and implementation of MAMS trials, statisticians, and MS clinicians, was tasked with generating design options for running an efficient, scalable, and flexible clinical trial by exploring different scenarios to determine the best design type. The group reviewed data from phase II and III randomized controlled PMS trials from January 1, 2009, to January 1, 2019, to inform key design measures for both stage 1 and 2 analyses, such as effect size, and the relationship between the interim and final outcomes.

To assess the statistical operating characteristics of the trial (e.g., type I and type II error rates), we simulated multiple trials with different correlation structures for the treatment effects on stage 1 and stage 2 outcomes, under different treatment effect assumptions. We also modeled the expected trial progress over time based on different assumptions (such as recruitment rates) and design parameters (such as sample size and treatment stopping rules) to the second stage. Further details of the simulation methods are reported in eAppendix 1 and 2 (links.lww.com/WNL/B900). To support the design of the stage 1 analysis, we analyzed brain atrophy data from the MS-STAT1^16^ and ASCEND^17^ clinical trials (for full Methods, see eAppendix 1, links.lww.com/WNL/B900). Modeling was conducted in Stata version 15 (StataCorp) and Microsoft Excel 2016.

### Patient and Public Involvement

A PPI strategy group was involved since the earliest conception of the project, including members of each of the expert consortium working groups. The PPI strategy group included 4 members of the MS Society Research Network (patients with MS) and the MS Society Public Involvement Officer. They contributed to discussions as the project developed and focused on ensuring that the research would meet the needs of the MS community. Additional workshops attended by a total of 34 people with MS held in 3 UK locations brought in further expertise of people with MS on topics including relevance, feasibility, and acceptability of all aspects of the trial design as well as recruitment and engagement strategies.

### Data Availability

Data not published in this article will be made available by request from any qualified investigator.

## Review of Previous Trials

Our review identified 15 eligible phase II (n = 8) and phase III (n = 7) randomized trials in PMS (Table 2). The median trial size was 374 participants (range 54–1,651) and median follow-up duration was 2 years (range 1–4.5 years). Trials included secondary progressive MS (SPMS) (n = 6), primary progressive MS (PPMS) (n = 6), and mixed PMS (n = 3). Confirmed disability progression on the Expanded Disability Status Scale (EDSS) was reported in 9 trials at different time intervals ranging from 3 to 6 months and a composite outcome was reported in 4. Two (29%) of the phase III trials (siponimod, ocrelizumab) and 4 (50%) of the phase II trials (ibudilast, lipoic acid, biotin, simvastatin) were positive for their primary endpoints.

**Table 2:**
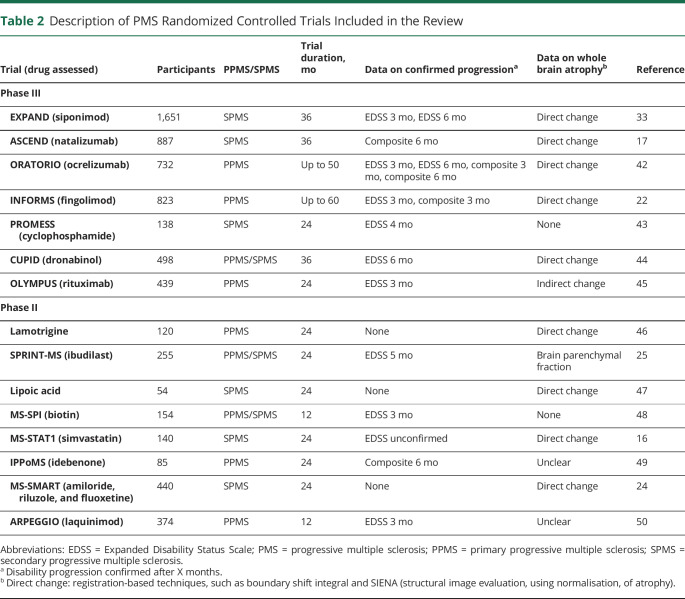
Description of PMS Randomized Controlled Trials Included in the Review

## Proposed MAMS Trial Design

### Overview

**Figure 1:**
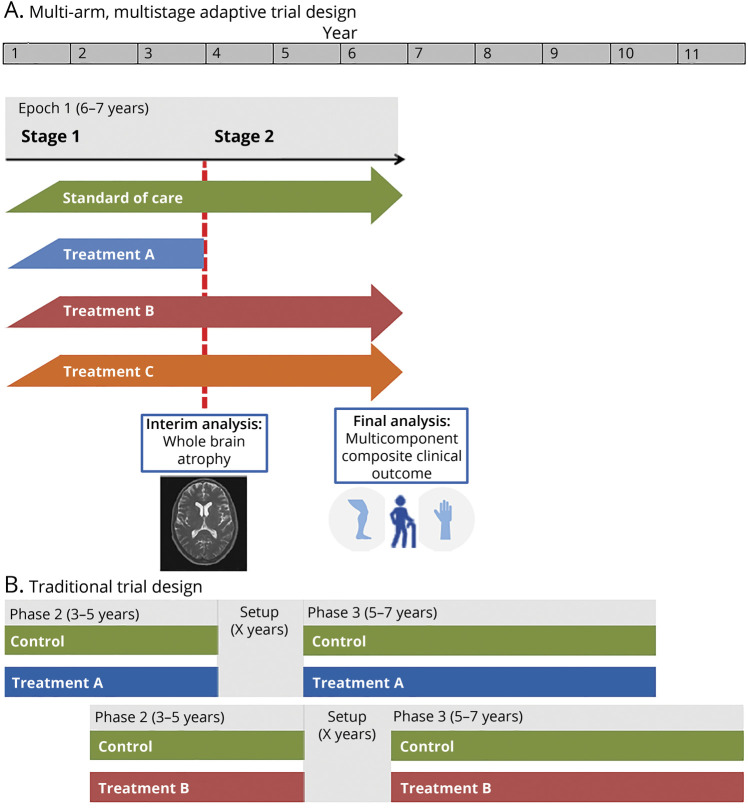
Schematic Representation of the MAMS Trial Comparing 3 Experimental Arms With Standard of Care in 2 Stages and Traditional 2-Arm Phase II and III Clinical Trials (A) Multi-arm multistage (MAMS) adaptive trial design. (B) Traditional trial design.

We propose the design of a 2-stage MAMS trial in PMS: 1 interim analysis to examine early evidence of treatment effect (stage 1) and 1 final confirmatory analysis of efficacy (stage 2). The trial would include 4 arms in stage 1: 1 standard of care (control) arm and 3 experimental arms. Any treatment that is sufficiently promising at the interim analysis will progress to stage 2, which will continue until the required number of events is reached, as represented in Figure 1.

It is expected that in the initial phase of the trial the standard of care arm for most participants will comprise best supportive care. Whereas ocrelizumab and siponimod have been approved for PMS, these treatments are not currently available to or suitable for all patients, in particular nonambulatory patients, who would be eligible for this proposed trial. If an efficacious therapy is subsequently found, this would then become the standard of care for future participants entering the platform.

The number of experimental arms was informed by feasibility constraints and the treatment selection group's work on number of repurposed therapies ready for clinical testing.^14^ Participants will be randomized with an equal probability between each of the 4 arms (1:1:1:1 ratio). In a standard multi-arm trial with *n* experimental arms, the optimal allocation ratio would be 
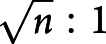
 in favor of the control arm. This is because the control participants contribute to each of the pairwise comparisons. However, for a MAMS trial, this depends on the number of arms continuing into stage 2, which is unknown.^18^ Unequal allocation would also make the trial less attractive to people with MS, as it results in a lower likelihood of being randomized to an experimental arm.

### Choice of Stage 2 (Final) Primary Outcome

The classic measurement tool and regulatory standard has been the EDSS,^19^ used to determine the time to disability progression. Its strengths and limitations are well-documented^20^ and numerous attempts have been made to evolve it, including using a composite measure based on progression in 1 or more of 3 endpoints: (1) increase in EDSS (of ≥1 point if baseline EDSS was <5.5 or ≥0.5 points if baseline EDSS was ≥5.5), (2) ≥20% increase in 9-Hole Peg Test (9HPT), or (3) ≥20% increase in Timed 25-Foot Walk (T25FW) (if ambulant).^21^

Composite measures achieve higher event rates than single measures (eAppendix 3, links.lww.com/WNL/B900), which can reduce trial duration and sample size. For example, in the INFORMS phase III trial of fingolimod in PPMS, >70% of participants had reached progression on the 3-month confirmed disability composite outcome by 3 years, as opposed to 50% based on EDSS alone.^22^ Inclusion of a measure of upper limb function also addresses the PPI group's interest in expanding the traditionally narrow EDSS inclusion criteria to include patients with higher levels of disability, to whom arm function is critical and measures of ambulation less relevant.^23^

Based on these considerations, we selected time to 6-month confirmed composite disability progression as the primary outcome for the final (stage 2) analysis. The composite outcome will be measured at baseline and every 6 months until the end of the follow-up. The time to progression will be from randomization until date of the initial disability progression (if subsequently confirmed). Based on earlier trials (eAppendix 3, links.lww.com/WNL/B900), we expect the rate of 6-month confirmed composite disability progression to be around 50% at 3 years.

### Choice of Stage 1 (Interim) Primary Outcome

Whole brain atrophy on MRI, measured as annualized percentage of brain volume change (PBVC), was selected as the primary interim outcome, based on the initial candidate drugs having primarily neuroprotective mechanisms of action. Brain atrophy reflects underlying neuroaxonal loss, which contributes to accrual of disability in PMS, and has been successfully used as a primary outcome in phase II trials, including MS-STAT1,^16^ MS-SMART,^24^ and SPRINT-MS.^25^ Importantly for a multistage trial, the treatment effect size on atrophy has been found to correlate with the clinical disability endpoint in a meta-analysis of relapsing-remitting MS (RRMS) trials.^24^

Methods of measuring PBVC include registration-based techniques (such as boundary shift integral and SIENA [structural image evaluation, using normalisation, of atrophy]) or brain parenchymal fraction, which quantify the amount of brain tissue contained within a contour surrounding the entire brain including CSF.^26^ Some therapies, particularly those with anti-inflammatory effects, can excessively reduce brain volume in the first months (pseudoatrophy),^27^ so it was recommended to assess PBVC also after at least 6 months on treatment.

We considered other imaging-based measures, including spinal cord atrophy, which contributes to MS disability progression and occurs at a faster rate than brain atrophy,^28^ neurite indices derived from diffusion MRI, which reflect the microstructural changes of axons and dendrites, and magnetization transfer imaging, which reflects demyelination and axonal loss.^19^ However, technical challenges limit widespread implementation and standardization across multiple centers.^27^ Although biofluid markers such as neurofilament light chain are associated in high concentrations with disability and brain atrophy,^29^ there are mixed findings on whether they are sensitive to treatment and they are not ready to be used as primary outcome measures until a validated, standardized, and widely accessible assay is available, with normative values of neurofilaments across age groups. Moreover, there is divergence of their utility in relapsing MS compared to PMS.^30,31^

### Predicted Brain Atrophy Rate

Nine of the reviewed trials reported a direct measure of change in whole brain volume (eAppendix 1, links.lww.com/WNL/B900). Brain atrophy rate varied between 0.4%/year and 0.7%/year in control arms. There was no clear pattern of differences between trials in PPMS or SPMS or by follow-up length. The SD for atrophy rate decreased with increasing follow-up length, ranging from 0.59%/year to 0.78%/year over 1 year, and 0.37%/year to 0.60%/year over 2 years.

**Figure 2:**
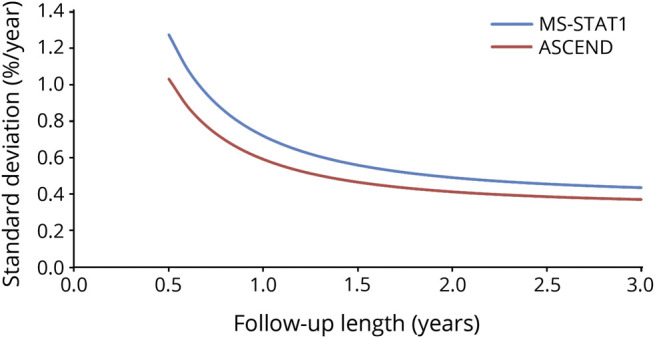
Predicted SD of Atrophy Rate for Varying Follow-up Length, Based on Modeling of MS-STAT1 and ASCEND Trial Data^20,35^

The predicted SDs based on applying our statistical model (eAppendix 1, links.lww.com/WNL/B900) to the data from MS-STAT1 and ASCEND are shown in Figure 2. The SD is expected to decrease rapidly with increasing length of follow-up, especially in the first 12 months. After 18–24 months, the reduction in SD becomes much smaller.

Timing of the interim analysis is an important consideration of adaptive designs. It should occur after accruing sufficient participant data to make a reliable decision on continuing or dropping treatment arms, but early enough relative to total trial recruitment to have value in informing adaptation of the trial design.^32^ Based on these considerations, PBVC after 18 months' follow-up will be assessed at interim analysis. This choice achieves a balance between reducing variance of the measure and ensuring that the interim analysis was sufficiently timely to make it worthwhile (see below). The SD at this point is predicted to be around 0.55%/year.

### Treatment Effects on Brain Atrophy and Clinical Progression

**Figure 3:**
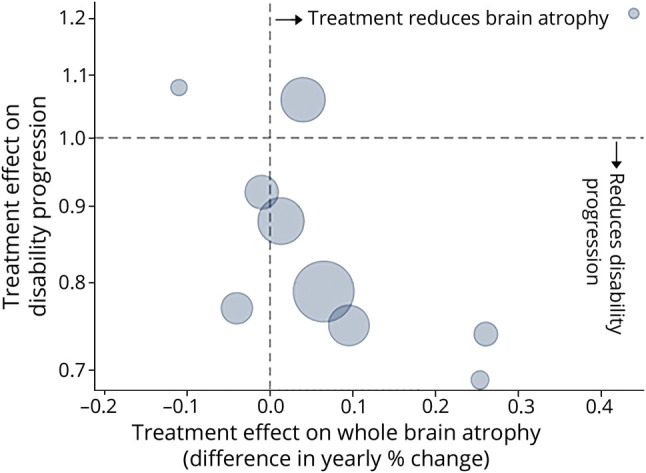
Association Between Treatment Effect on Brain Atrophy and Disability Progression in Progressive Multiple Sclerosis Trials The size of each circle is proportional to the trial size.

A key criterion for the stage 1 outcome is the ability to identify treatments expected to be ineffective and also potentially effective in terms of the final (stage 2) outcome. We reviewed trials reporting treatment effect on both brain atrophy rate and clinical progression. Trial results are reported in eAppendix 2 (links.lww.com/WNL/B900) and summarized in Figure 3. There is a negative correlation, indicating that drugs with a stronger effect on reducing brain atrophy in PMS were more effective on clinical outcomes, confirming findings in RRMS.^26^

Our trial targets a treatment effect of 25% relative reduction in the 6-month confirmed disability progression rate, that is, a hazard ratio of 0.75. This is a clinically important effect in slowing progression in ambulation, upper limb function, or disability, which has been achieved in previous trials.^33^ Assuming 50% of patients experience a disability progression by 3 years in the control arm, a 25% relative reduction would equate to a 12.5% absolute difference (50% control vs 37.5% active treatment).

Based on the review of previous trials, we assumed an effective treatment would reduce the rate of whole brain atrophy by around 0.15%/year, from 0.55%/year to 0.40%/year.

### Stage 1 Sample Size

The sample size for stage 1 analysis was based on pairwise comparisons of whole brain atrophy rate at 18 months between each intervention arm and standard of care. A 1-sided test is used for the interim analysis, with a treatment continuing to the second stage if there is evidence in favor of a lower atrophy rate compared to standard of care. We chose 95% power because a priority of the interim analysis is to minimize the chance of stopping an arm when the treatment is genuinely active in slowing brain atrophy (i.e., avoid false-negatives). Stage 1 alpha (type I error rate) captures the probability of an ineffective treatment to continue to the second stage. It should be chosen to balance minimizing this risk while ensuring the timeliness of the interim analysis. Designs were considered with stage 1 alpha between 20% and 50% with the final choice of 35% representing an achievable sample size and timely interim analysis (see trial timeline). This is in line with other MAMS trials,^15^ but differs from the 5% commonly used in confirmatory analysis, as the objectives here are different. Assuming an SD of 0.55%/year (see above), 111 observations per arm will allow 95% power to detect a 0.15%/year difference at a 1-sided significance level (alpha) of 35%. Allowing for 10% drop-out, 123 participants are needed per arm.

Therefore, we recommended that stage 1 analysis be conducted once 18 months' brain atrophy data are available for 111 participants per arm, with pairwise comparison for each experimental arm compared to the control arm. If the 1-sided *p* value is below 0.35, then the treatment arm is continued into stage 2.

### Stage 2 Sample Size

The sample size for the stage 2 analysis was based on comparing the time to confirmed disability progression between each intervention arm to standard of care. For each pairwise comparison, to have 90% stage 2 power to detect a hazard ratio of 0.75 at the 2-sided stage 2 significance level of 5% (or equivalently a 2.5% 1-sided significance level), 281 progression events are required in the control arm and 600 participants per arm are needed.

The stage 2 significance level was set at a 2-sided 5% level, as in standard confirmatory trials, corresponding to a 1-sided level of 0.025. The question of multiplicity (adjusting significance level due to multiple comparisons) has been discussed before in MAMS.^18^ We aimed to select drugs with different mechanisms, which might be viewed as independent evaluations, similar to multiple trials being conducted,^34^ and therefore did not apply any correction for multiple comparisons. If drugs of similar action are selected (e.g., different doses of the same drug), an appropriate correction (e.g., Dunnett^35^) should probably be applied. The statistical power in a time-to-event analysis is determined by the number of events. Recruiting 600 participants per arm should be sufficient to observe the required 281 progression events in the control arm in a timely manner. This number of events is anticipated to occur around 18 months after the last participant has been enrolled, assuming a 10% drop-out rate and 50% disability progression rate by 3 years and recruitment rate, as described in eAppendix 4 (links.lww.com/WNL/B900) (see trial timeline).

### Trial Operating Characteristics

We conducted simulations to assess the operating characteristics of the proposed trial design under different scenarios (see eAppendix 5, links.lww.com/WNL/B900, for Methods and full Results). Table 3 shows the overall trial characteristics, depending on the number of truly effective treatments at the start of the trial. In all scenarios, the probability to wrongly conclude that 1 or more treatments is effective (false-positive) is below 4%. The chance of correctly concluding that at least 1 treatment is effective (power) if a single effective drug enters the trial is around 87%, but this increases to above 96% if more than 1 effective drug enters the trial.

**Table 3:**
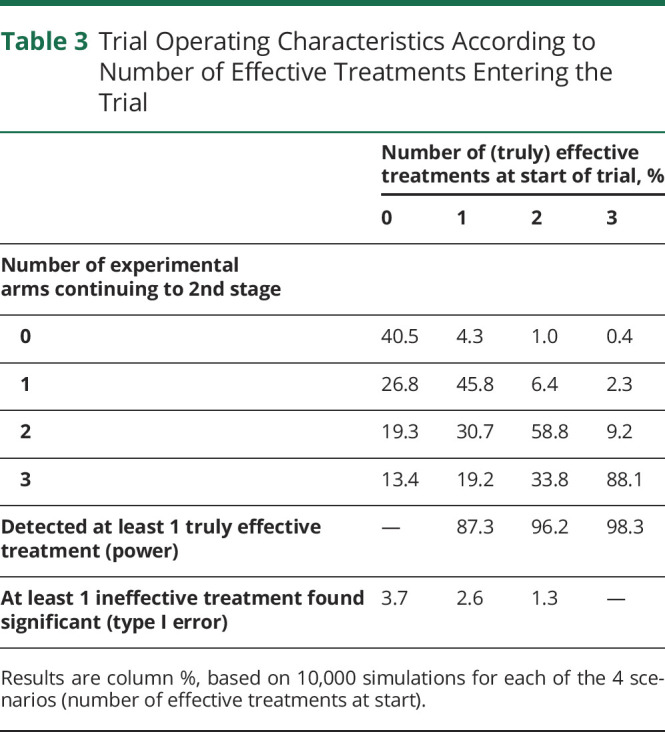
Trial Operating Characteristics According to Number of Effective Treatments Entering the Trial

### Trial Timeline

An important consideration in adaptive trials is to anticipate the possible dynamics of the trial over time, including the relative timing of the interim and final analyses. eAppendix 4 (links.lww.com/WNL/B900) describes the assumptions made and how the timeline was modeled. Results are summarized in Table 4. Under a base case scenario of 40–50 participants recruited per month and 1 experimental arm continuing into stage 2, we expect the interim analysis to be conducted after around 3.4 years, and the final analysis after 6.1 years (ranging between 5.7 and 6.6 years depending on different scenarios modeled).

**Table 4:**
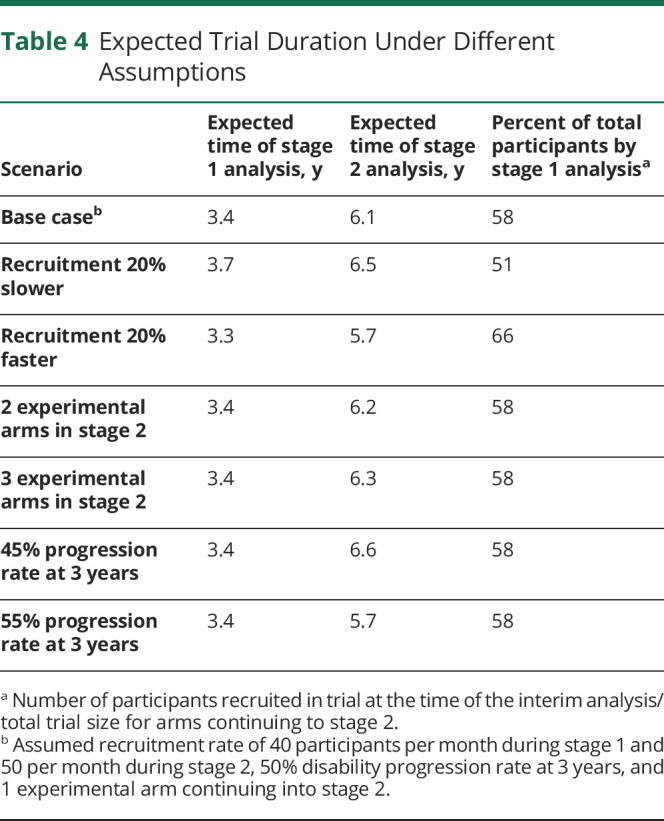
Expected Trial Duration Under Different Assumptions

## Discussion

MAMS trials have considerable potential in PMS, where there are many candidate therapies, as well as relevant interim outcome measures that have appropriate relationship to final clinical outcomes. We propose a MAMS trial design that could potentially accelerate the evaluation of new treatments in PMS.

### Advantages

The proposed MAMS design leads to efficiencies in both sample size and trial duration compared to traditional separate phase II and III trials of single treatments. A single control arm is used to assess multiple experimental arms and participants recruited in stage 1 seamlessly continue to be included in the stage 2 analysis without additional setup time in between. This trial is expected to last 6–7 years with 1,900 participants, encompassing the stage 1 and 2 evaluations of 3 initial candidate treatments (Figure 1 and eAppendix 4, links.lww.com/WNL/B900).

In comparison, under the traditional approach, around 630 patients would be required in each of 3 phase II studies to have 90% power with 5% type I error under the same assumptions. If one of these treatments was found to be effective and proceeded to phase III, 1,200 additional participants would be required, totaling 3,090 participants. Separate phase II and III trials of a single treatment would be expected to take more than 10 years, with 3–5 years for phase II, 5–7 years for phase III, and additional setup time between the two (Figure 1). For example, evaluation of high-dose simvastatin is following a more conventional path with separate phase II (MS-STAT)^16^ and phase III (MS-STAT2)^36^ trials. Recruitment to MS-STAT started in 2011 and MS-STAT2 is expected to be completed by 2025, which corresponds to 14 years overall.

### Challenges

Planning and setting up a MAMS adaptive platform trial is considerably more complex than standard phase II and III trials and may take up to 12 to 18 months. In particular, statistical simulations examining different design options, scenarios, and parameters are essential to optimize efficiency and select appropriate trial operating characteristics while preserving the overall integrity of the trial. The initial modest investment in time and resources will be further offset by shorter subsequent setup times for further treatments added to the platform.

As adaptive platform designs are relatively novel in neurodegenerative diseases, there is a perception that regulatory agencies may not immediately accept them as equal to more conventional phase III studies. However, a precedent has been set for regulatory approval of MAMS platform trials in settings such as oncology^7^ and infectious diseases^8^ and our experience in these other disease areas suggests that regulators are becoming more open to, and knowledgeable and welcoming about, such designs.

### Patient and Public Involvement

The PPI group actively participated in the entire trial design process, as well as treatment and outcome measure selection, to ensure the needs of people with MS were being met. For example, it was important, particularly for nonambulatory people with MS, to include an assessment of upper limb function in the primary efficacy endpoint and proposed secondary outcomes included patient-reported outcome measures of key symptoms such as fatigue. Feedback indicated the trial design was well-received and acceptable, despite being more complex. Perceived advantages included the ability to evaluate multiple candidate treatments and the relatively lower likelihood of randomization to placebo. If a participants' treatment arm is discontinued after interim analysis, there is the potential opportunity to re-enter the trial in a continuing arm or future trials in the platform. Some participants expressed concern about the total trial duration, but this was offset by the favorable consensus overall regarding potential acceleration of treatment discovery.

### Scope and Future Developments

This article is based on work conducted by the trial design working group and in many senses is an evolution from our work carried out a decade ago.^37^ A program grant proposal based on activity of all working groups of the UK MS Society's Expert Consortium for Progression in MS was submitted to the UK MS Society in November 2019 and received favorable international peer and lay review. Funding has been awarded to develop the protocol and deliver the first active arms plus standard of care in the MAMS trial platform, with recruitment expected to commence in 2022. Whereas this article focuses on evaluation of only the first 3 candidate therapies, the adaptive MAMS platform will allow addition of new treatment arms^7^ and re-randomization of participants from discontinued arms in the future.^38^ Drugs with predominantly remyelinating potential will likely require additional and alternative endpoints at the interim analysis stage. Further aspects of the trial protocol, for example secondary and exploratory outcomes and recruitment infrastructure, are beyond the scope of this article.

### Adaptive Platform Trials in Other Neurologic Disorders

Like PMS, conditions such as Parkinson disease, Alzheimer disease, and motor neuron disease are increasing in prevalence, have significant impact on patients, carers, and health care systems, and have no or few therapies that slow or prevent progression. An improved understanding of disease pathophysiology in recent years has led to a growing pipeline of potential therapeutics. For example, a 2020 review identified 121 agents in 136 phase I to III clinical trials for Alzheimer disease, with an increasing number of disease modification treatment candidates over the past 5 years.^39^ However, these conditions face similar challenges of efficiently translating candidate drugs into effective treatments with many disappointing phase III clinical trial results to date. Various reasons for this have been proposed, including the need to improve trial design.^10,12,40^

MAMS designs are particularly relevant when there are multiple candidate therapies to be trialed and when a reliable early marker of clinical efficacy is available. MAMS adaptive platform trials have been planned and initiated to accelerate successful drug discovery in these disorders. The Motor Neuron Disease–Systematic Multi-arm Adaptive Randomization Trial (MND-SMART) will initially test 2 repurposed drugs against a common placebo.^11^ The Dominantly Inherited Alzheimer's Network Trials Unit (DIAN-TU) platform trial established in 2012 was a multi-arm trial of 2 anti-amyloid monoclonal antibodies. Although neither drug met the primary cognitive endpoint,^41^ lessons learned, including refinements in participant and outcome measure selection and trial duration, have led to several emerging platform trials, such as the AHEAD study evaluating different doses of an anti-Aβ monoclonal antibody in 2 phase III clinical trials that respectively use amyloid PET and cognitive testing as the primary outcome measures.^9^

The principles of designing a PMS MAMS trial as outlined in this article are relevant to other neurodegenerative conditions, but each condition will present unique considerations and challenges, including selection of biologically and clinically relevant, sensitive, and timely interim and final outcome measures, determination of the most appropriate patient population for inclusion, and trial duration required to detect a meaningful effect.

We propose a design for a MAMS trial in PMS for evaluation of 3 repurposed neuroprotective drugs compared to standard of care. Although more complex in design, efficiencies in participant numbers and trial duration, as well as the ability to incorporate adaptive elements and continually test newly identified treatments through an ongoing platform, make this approach more likely to succeed in finding effective therapies that target disability progression in PMS in a timely manner.

## Glossary

9HPT: 9-Hole Peg Test
EDSS: Expanded Disability Status Scale
MAMS: multi-arm multistage
MS: multiple sclerosis
PBVC: percentage of brain volume change
PMS: progressive multiple sclerosis
PPI: patient and public involvement
PPMS: primary progressive multiple sclerosis
RRMS: relapsing-remitting multiple sclerosis
SPMS: secondary progressive multiple sclerosis
T25FW: Timed 25-Foot Walk
